# Pregnant and breastfeeding women’s intention to follow medical advice before antibiotic use: a comparative pilot analysis using the theory of planned behavior in Mahajanga, Madagascar

**DOI:** 10.1186/s12889-026-26190-1

**Published:** 2026-01-12

**Authors:** Daouda Kassié, Emilie Dama, Timothée Razafindrabesa, Aina Harimanana, Voahirana Ravololomihanta, Isidore Juste Bonkoungou, Elliot Fara Nandrasana Rakotomanana

**Affiliations:** 1 Institut Pasteur de Madagascar, Antananarivo, 101 Madagascar; 2 CIRAD, UMR ASTRE, Antananarivo, 101 Madagascar; 3 ASTRE, CIRAD, INRAE, Université de Montpellier, Montpellier, France; 4 Division of Global Health Protection, Country Office, Center for Global Health, US Centers for Disease Control and Prevention, Ouagadougou, Burkina Faso; 5 Direction de la santé Familiale, Ministère de la santé Publique de la République de Madagascar, Antanarivo, 101 Madagascar; 6 Department of Biochemistry and Microbiology, Université Joseph KI-ZERBO, Ouagadougou 03, Ouagadougou, Burkina Faso

**Keywords:** Women, Medical advice, Antibiotic, Theory of planned behavior, Madagascar

## Abstract

**Background:**

Antibiotic misuse among pregnant and breastfeeding women is a critical public health challenge in low-resource settings. Individual attitudes, social expectations, and structural barriers shape women’s intentions to follow medical advice. Thus, this study investigates how attitudes, subjective norms, and perceived behavioral control shape pregnant and breastfeeding women’s intentions to seek medical advice prior to antibiotic use.

**Methods:**

Using the theory of planned behavior (TPB), this mixed-methods study collected data from 115 women in the urban and rural districts of Mahajanga, Madagascar. Qualitative analysis explored statements and informational practices, whereas linear regressions, partial least squares regressions (PLS-R), and binary logistic regression (BLR) identified determinants of intention.

**Results:**

Overall, 79.1% of women expressed a willingness to consult healthcare providers, although this was often undermined by unclear prescriptions, family influence, and limited access to care. In urban areas, subjective norms (SN) were the strongest predictor of intention, reflecting the role of social expectations. In rural areas, perceived behavioral control (PBC) was most influential, underscoring the importance of access and self-efficacy. Logistic regression confirmed these patterns: favorable attitude (ATB) (*p* = 0.005) and high PBC (*p* = 0.029) significantly increased intention in rural areas. Obtaining antibiotics from official sources was associated with a significantly lower intention in rural areas (*p* = 0.037).

**Conclusion:**

Strengthening women’s intention to follow medical advice requires addressing knowledge gaps, improving communication, and reducing structural barriers. Interventions should be focused on access and empowerment in rural areas and education and social norms in urban areas. In both urban and rural areas, the engagement of community health workers is a trusted mediator. These findings highlight the need for interventions that integrate both behavioral and structural approaches to ensure rational antibiotic use in terms of maternal and child health.

**Supplementary Information:**

The online version contains supplementary material available at 10.1186/s12889-026-26190-1.

## Background

The administration of antibiotics to pregnant or breastfeeding women may result in the transfer of the drug and its metabolites into breast milk, potentially disrupting the infant’s gut microbiome and increasing their vulnerability to future infections and the transmission of antibiotic resistance genes [1]. Adherence to medical advice is therefore a critical determinant of effective public health interventions, especially in disease prevention, chronic care, and antimicrobial resistance control [2]. In many settings, adherence depends less on the availability of services than on individuals’ willingness to follow recommendations. Nonadherence may stem from misunderstanding instructions, a lack of confidence in efficacy, or a fear of side effects, often leading to premature discontinuation.

Beyond individual-level barriers, psychosocial constructs strongly influence treatment decisions. Perceived social norms and behavioral control, which are core elements of the theory of planned behavior (TPB), are particularly important for such decisions. Several theoretical models have examined adherence to medical advices, including the health belief model [3], social cognitive theory [4], protection motivation theory [5], the theory of reasoned action, and the TPB [6]. Among these, the TPB provides a validated framework based on attitudes, subjective norms, and perceived behavioral control, shaping the immediate antecedent of behavior [7, 8] and is widely applied to vaccination, medication adherence, and preventive health [9, 10]. Unlike other behavioral frameworks, the Theory of Planned Behavior (TPB) conceptualizes intention as the proximal determinant of action. For health-seeking behaviors—including antibiotic use, consultation of health professionals, and medication purchasing—intention has consistently been shown to be the strongest predictor of behavior, as illustrated by studies on antibiotic prescribing decisions among physicians [11–13] and on pregnant women’s intentions to use institutional delivery services and their predictors in Ethiopia [14]. These empirical findings support the relevance of TPB for understanding women’s intentions to follow medical recommendations in Madagascar.

In Madagascar, urban–rural inequalities in living conditions and access to health care are particularly pronounced. The majority of the population lives in rural areas characterized by widespread poverty, lower health-care provider density, long distances to health facilities that are often difficult to reach year-round, and more limited health-care infrastructure compared with urban areas [15]. Moreover, previous epidemiological studies in Mahajanga documented a high burden of infectious diseases, highlighting the pressing need for adherence to medical recommendations [16, 17]. In this context, factors such as place of residence (urban vs. rural), distance to health facilities, level of education and socioeconomic status, exposure to mass media, and residence in communities with higher literacy levels reflecting stronger subjective norms have been shown to be key determinants positively associated with the use of maternal health services in Madagascar, particularly facility-based delivery [18]. It is therefore plausible that the determinants of women’s intention to follow medical recommendations prior to antibiotic use are not structured in the same way in urban and rural areas.

In Madagascar, antibiotic misuse among pregnant and breastfeeding women is a growing concern. Our previous study using the Q-method among pregnant and breastfeeding women in both urban and rural settings in the Mahajanga region already identified distinct profiles of perceptions related to antibiotic use according to place of residence, educational level, and reproductive trajectories. These groups included women who strictly adhered to medical instructions and recommendations, and women whose beliefs about antibiotic use were shaped by information from multiple sources, including healthcare professionals, personal experience, or advice from nonmedical relatives. A third group comprised women who could not be clearly classified into either of the two profiles [19], but evidence on the psychosocial drivers of decision-making remains largely unexplored. This observed heterogeneity justifies conducting a stratified urban–rural analysis of behavioral determinants in the present study, rather than assuming a single model applicable to the entire study population. To our knowledge, no other prior studies have systematically compared women’s intention on the use of antibiotics in urban and rural areas, nor they have applied robust analytical triangulation. Thus, our study addresses these gaps by applying the TPB within a mixed-methods between urban and rural contexts. The objective is to identify the psychosocial and sociodemographic determinants of pregnant and breastfeeding women’s intention to seek or not medical advice before antibiotic use in Mahajanga, Madagascar.

## Methodology

### Study sites

**Fig. 1:**
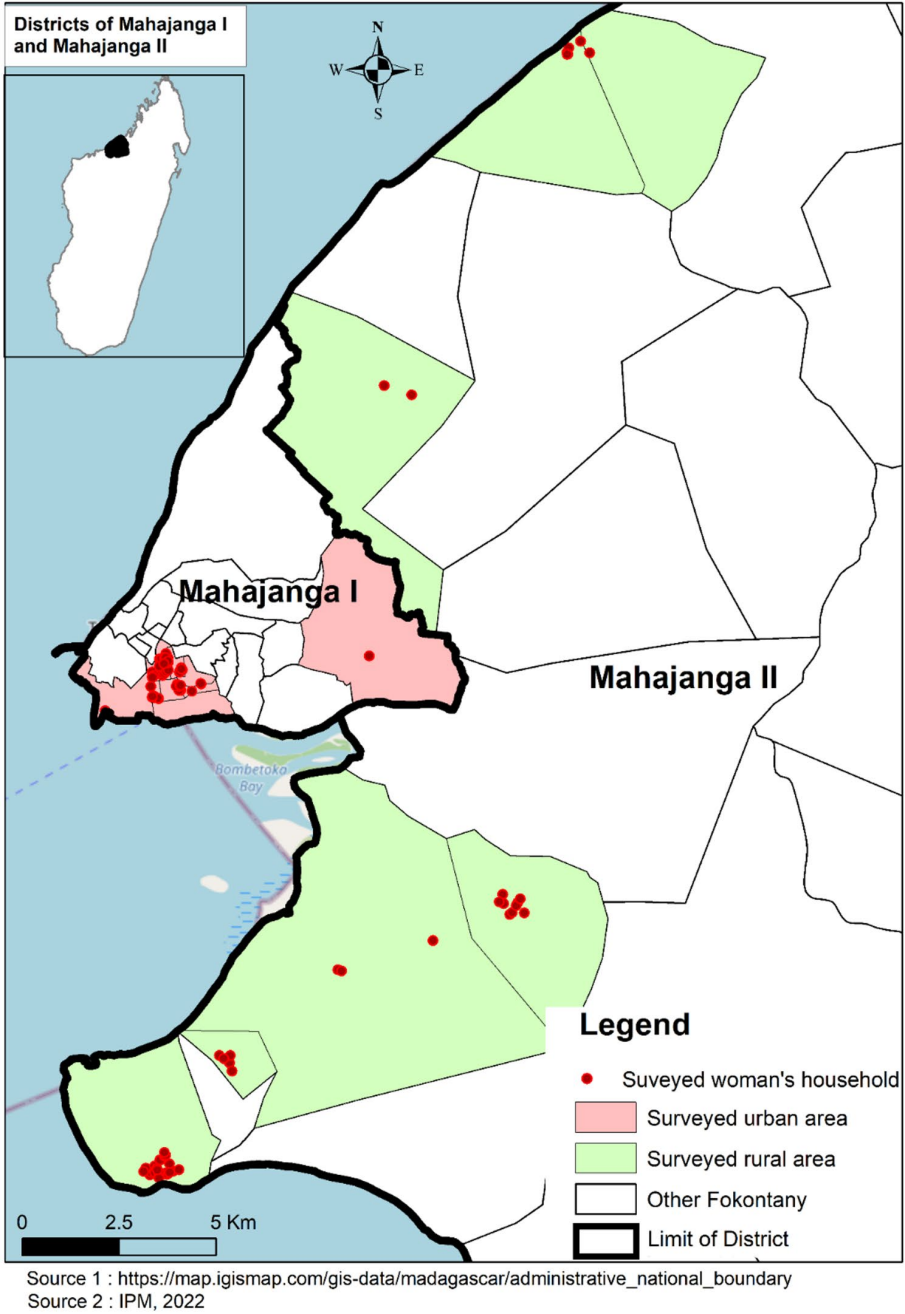
Study area and the women investigated location in urban and rural areas

This study was conducted in northwestern Madagascar, which has clear sociodemographic differences between urban and rural areas. Mahajanga I with a population estimated at 333,096 is the regional capital, characterized by the urban setting, with an urbanization rate nearly twice the national average (35.8% vs. 19.3%). In contrast, Mahajanga II which surrounds the city is predominantly rural with an estimated population of 596,296). According to the 2018 census, the region’s female population growth rate was 3.4%, which is above the national rate of 3.04% [20]. Data were collected in Mahajanga I between June and July 2022 and in Mahajanga II between August and September 2022. In each district, one *Fokontany*, the smallest administrative unit, was randomly selected [Fig. 1].

### Study design

This prospective mixed-methods study was grounded in Q-methodology and the TPB. Q-methodology allows the systematic exploration of subjective viewpoints by combining structured sorting of statements with qualitative interviews. Prior to data collection, a pilot exercise involving eight women was conducted to test the clarity of the statements. These participants were excluded from the final sample.

### Q-methodology

Q-methodology is a mixed-methods approach designed to systematically explore subjective viewpoints. It combines qualitative and quantitative techniques, using factor analysis to group individuals according to how they rank and interpret a set of statements about a given topic [21]. Unlike conventional survey research, Q-methodology does not require a priori statistical sample size calculations because its objective is not population inference but rather the identification and structuring of subjective viewpoints. Sample adequacy is therefore assessed in terms of viewpoint saturation and factor stability, rather than representativeness. Consequently, large samples are not necessary, and participants are strategically selected using purposive sampling to ensure that the diversity of potential viewpoints is adequately represented (Alderson 2018). As methodological guidance suggests, sample sizes ranging from 20 to 60 participants are typically sufficient for most Q studies [22–24]. Previous Q-methodology studies indicate that approximately 30 to 80 statements are generally sufficient to capture the breadth of perspectives within a debate while maintaining feasibility for participants [24, 25]. Larger samples do not violate the methodological principles of Q methodology and may be particularly useful when the research aims to examine subgroup differences or capture greater heterogeneity of perspectives.

### Theoretical framework

**Fig. 2:**
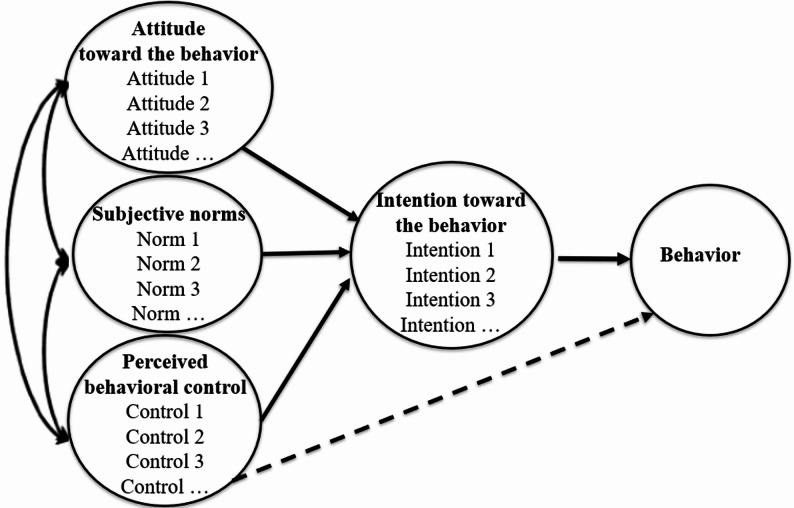
Theory of planned behavior framework

This study was guided by the Theory of Planned Behavior (TPB) [7] [Fig. 2]. According to this theory, an individual’s behavior is primarily determined by their intention to perform it, as well as by perceived behavioral control when the behavior is not entirely under voluntary control. In our study, the behavior refers to follow medical advice before antibiotic use. This theory posits that intention depends on three key constructs:

- Attitude toward the behavior (ATB), which is defined as an individual’s favorable or unfavorable evaluation of the behavior (e.g., “*The consumption of antibiotics causes diseases in mothers and children*”).
- Subjective norms (SN), which refer to the perceived social pressure to perform or not perform the behavior (e.g., “*Healthcare providers always communicate the possible effects of antibiotics*”).
- Perceived behavioral control (PBC), which refers to an individual’s perception of their ability to carry out the behavior (e.g., “*Pregnant and breastfeeding women can take antibiotics in their own way*”).
- Behavioral intention (BI), which is the conscious motivation to act (e.g., “*If I take antibiotics before my delivery*,* I inform the doctor*”).

According to TPB, ATB, subjective norms, and perceived behavioral control directly influence intentions. Additionally, perceived behavioral control has a direct effect on behavior [Fig. 2]. This theory further suggests that the more favorable the attitude and subjective norms are and the greater the perceived control is, the stronger the individual’s intention to engage in the behavior. The relationship between intention and behavior indicates that people are more likely to perform behaviors they intend to undertake. Finally, the association between perceived control and behavior suggests not only that individuals are more likely to adopt behaviors over which they perceive some degree of control but also that they may be constrained or prevented from enacting behaviors they consider to be beyond their control [7].

Although this Theory of Planned Behavior (TPB) analysis draws on the same interview material as the Q-methodology study, the objectives of the two approaches differ. Q-methodology was used to identify shared subjective viewpoints among the participants. Thematic analysis was subsequently conducted to provide a theory-guided interpretation of some of these viewpoints that are relevant to public health by linking participants’ narratives to TPB constructs. The two methods are therefore complementary: Q-methodology structures the typology of perspectives, while thematic analysis deepens their theoretical interpretation.

### Study population

A total of 115 pregnant or breastfeeding women were recruited through convenience sampling, which is consistent with Q-methodology exploratory work principles that prioritize the diversity of perspectives rather than demographic representativeness [23]. Eligibility criteria included being pregnant or breastfeeding and age ≥ 14 years (the lower age of early pregnancy in the context of Mahajanga), pregnant or breastfeeding women, use of at least one antibiotic in the previous three months, and provision of informed consent. Community health workers supported recruitment.

### Data collection and processing using Q-methodology

A concourse of subjective opinions on antibiotic use was developed from the literature, and field observations (Q-set). From this, 36 representative statements were selected to form the Q-sample, translated in Malagasy, and pretested. Participants sorted the 36 items on a quasinormal grid ranging from “most agree” (+ 3) to “most disagree” (− 3). Sorting was conducted individually with trained interviewers, who also probed for explanations of extreme choices. This approach integrated quantitative rankings with qualitative explanatory narratives in a previous study [19]. We balanced these statements across the TPB constructs.

### Data management and item scoring

For TPB analyses, we applied a simple linear transformation to express responses on a conventional 1–7 Likert scale, ensuring that higher scores consistently indicate more positive ATB or stronger BI. This type of recoding of bipolar scales is consistent with TPB questionnaire guidelines and with previous TPB applications that rescale − 3 to + 3 formats into Likert scale [8]. Likert scale has been used by Ayana et al. (2020) to predict pregnant women intention to use institutional delivery service using TPB [14]. This is a simple linear transformation, that does not affect the pattern of associations among variables like correlations and regression coefficients, but facilitates the computation and interpretation of TPB composite scores. Composite mean scores were calculated for each TPB construct, then used as continuous quantitative variables in multiple linear regression (MLR) and partial least squares regression (PLS-R), with the intention score (BI) as the dependent variable. The results of the PLS-R analysis are reported in the Supplementary Materials [Supplementary results PLSR].

### Qualitative grouping and coding of items into TPB constructs

Each statement was assigned to a TPB constructs through semantic analysis of linguistic markers (cognitive, affective, normative, or modal). Data were coded independently by the two first co-authors, and discrepancies were reconciled with two other co-authors. This deductive, theoretically stratified approach to Q-set development is widely applied in Q-methodology research [23, 26]. This process resulted in 12 items for attitude, 8 for subjective norms, 11 for perceived behavioral control, and 5 for intention [Supplementary Table S1].

### Qualitative analyses of semi-structured interviews

Semi-structured interviews during Q-sorting explored participants’ reasoning, particularly for extreme placements (+ 3 and − 3) [Supplementary File S2]. Interviews were recorded in Malagasy on tablets and managed with Kobo Toolbox^®^. The data were transcribed, translated into French then English. Transcription and translation followed a multi-step validation process. Interviews were first transcribed verbatim in Malagasy and translated into French by trained bilingual research assistants. The French versions were then translated into English by a second bilingual researcher. To ensure conceptual equivalence across languages, all translations were independently reviewed by two senior members of the research team fluent in Malagasy, French, and English. This multi-stage verification approach is commonly recommended in multilingual qualitative research to maintain data quality and validity [27–29]. The data were thematically analyzed with Nvivo© (version 1.7.1) using a coding guide based on TPB constructs to ensure consistency [8]. Q-methodology and thematic analysis were complementary. in fact, Q-methodology was used to identify and structure shared belief profiles based on participants’ Q-sorts, whereas thematic analysis was employed to deepen the interpretation of these profiles by elucidating themes, meanings derived from participants’ narratives. In the present study, verbatim excerpts were selected to illustrate each TPB construct, emphasizing theoretical alignment and adding explanatory depth to the quantitative results. This strategy aligns with the methodological guidance for integrating qualitative and quantitative components in TPB-based research [30, 31].

### Quantitative data analyses

All analyses were conducted using R (v.2025.05.0). Urban (Mahajanga I) and rural (Mahajanga II) datasets were analyzed separately. Associations between sociodemographic variables (age, marital status, education, employment, gravidity, breastfeeding status, and timing/source of antibiotic use) and area of residence were tested using chi-square tests (*p* ≤ 0.10 for trends; *p* ≤ 0.05 for significance). Bold p-values in the tables indicate significance at the 0.1 level.

Multiple linear regressions (MLR) were performed for each area: Model 1 included TPB constructs, and Model 2 additionally adjusted for age, gravidity, and household size. Multicollinearity was assessed using the variance inflation factor (VIF > 5). Associations were evaluated using β coefficients, p values, and explained variance (R²), with β ≥ 0.3 and *p* ≤ 0.05 indicating strong predictors.

Binary logistic regression (BLR) used dichotomized behavioral intention (BI) scores (median split per site) as the outcome variable. ATB, SN, PBC, and sociodemographic variables were used as explanatory variables for BI. Multivariable were performed using Stepwise selection (backward AIC) identified the predictors. The results are reported as adjusted odds ratios (aORs) with 95% CIs (*p* ≤ 0.05). Model fit was assessed using the Hosmer–Lemeshow test and discrimination with the area under the ROC curve (AUC).

**Fig. 3:**
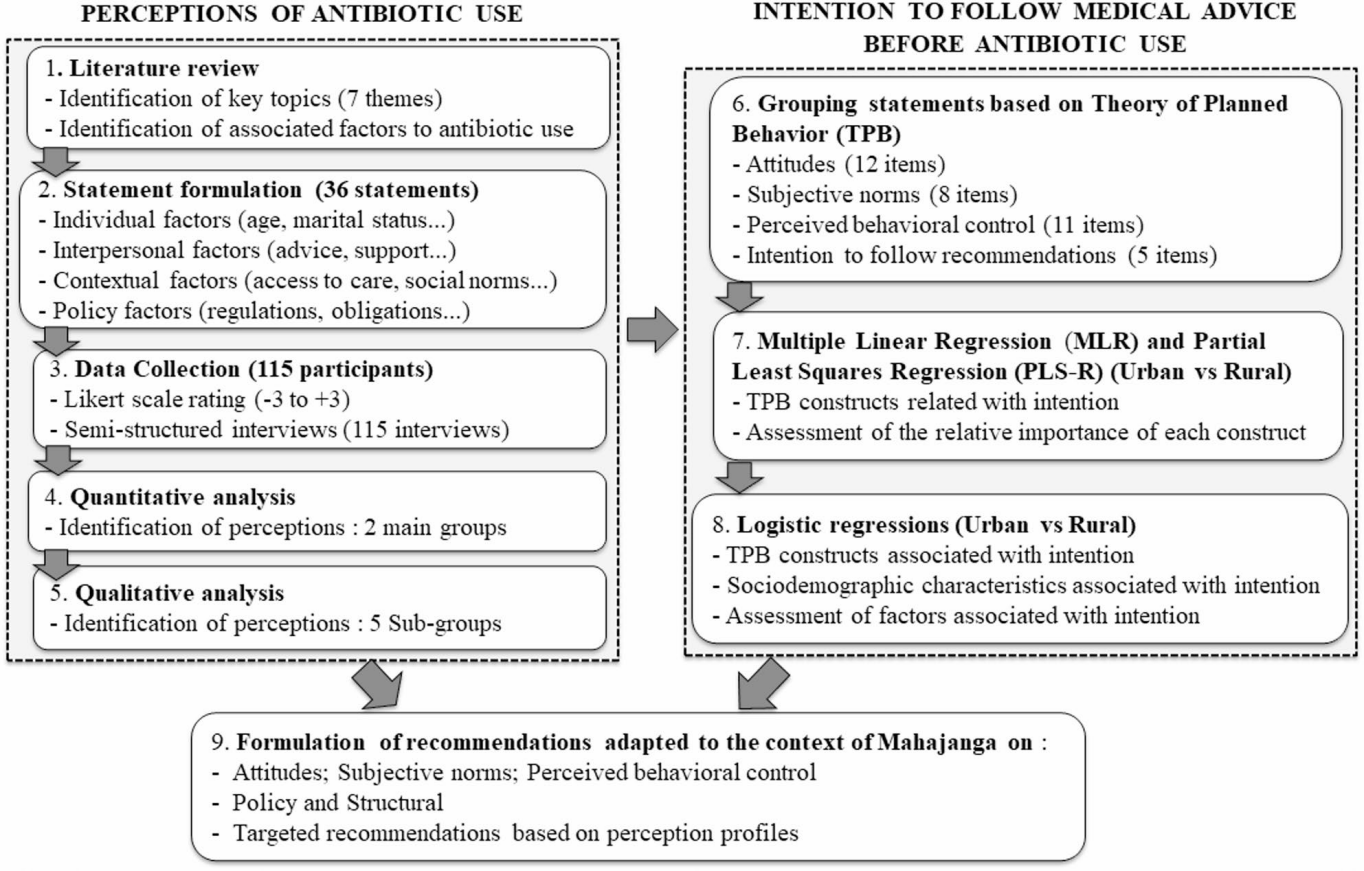
Study flowchart for women’s perceptions of antibiotic use, intentions to follow medical advice and final recommendations

The study design, women’s perceptions of antibiotic use, their intention to follow medical advice, and the resulting recommendations are summarized in Fig. 3.

## Results

### Sociodemographic characteristics of the study participants

Among the 115 women included in the study, 87.83% were breastfeeding, with no significant difference across the groups. Urban participants were more likely to be older than 27 years (33.04%) and more educated (46.09% reached secondary education or higher). Rural participants were predominantly younger (30.44% less than 27 years). Employment patterns varied significantly across areas: urban women were more frequently engaged in trade (22.60% vs. 8.70%), whereas rural women were more often involved in diverse activities, such as fishing, hairdressing, and tailoring (26.96% vs. 9.57%). Unemployment or lack of professional activity remained high overall (32.17%, *p* < 0.001) [Table 1].

**Table 1:** Sociodemographic features of the study participants by place of residence

Variables / Area	Pearson Chi^2^ test *p*-value	Urban		Rural		Total	
		*n*	%	*n*	%	*n*	%
Woman status	0.337						
Breastfeeding		54	46.96	47	40.87	101	87.83
Pregnant		5	4.35	9	7.84	14	12.17
Age category	**0.0069**						
≥ 27 years		38	33.04	21	18.26	59	51.30
< 27 years		21	18.26	35	30.44	56	48.70
Marital status	**0.0903**						
Married		54	46.96	44	38.26	98	85.22
Unmarried		5	4.35	12	10.43	17	14.78
Educational level	**0.0390**						
At least secondary level		53	46.09	41	35.65	94	81.74
Under secondary level		6	5.22	15	13.04	21	18.26
Person who recommended antibiotics used	0.5858						
Health agent		45	39.13	46	40.00	91	79.13
Other persons		14	12.17	10	8.70	24	20.87
Employment	**0.0001**						
Other activities		11	9.57	31	26.96	42	36.53
Trader		26	22.60	10	8.70	36	31.30
No professional activity		22	19.13	15	13.04	37	32.17
Antibiotic consumption period of use	**0.0620**						
≤ 1 month		29	25.22	17	14.78	46	40.00
2–3 months		30	26.09	39	33.91	69	60.00
Gravidity	0.8027						
≤ 2		34	29.57	30	26.09	64	55.65
≥ 3		25	21.74	26	22.61	51	44.35

Overall, 60% of the participants reported antibiotic use over the past two to three months. Urban women were more likely to report use in the past month (25.22% vs. 14.78%), whereas rural women more often reported use two to three months prior (33.91% vs. 26.09%, *p* = 0.062). [Table 1].

### Qualitative analysis of women’s intention to follow medical advice

The BI was particularly strong when women clearly perceived the risks of self-medication due to the fear of overdosing and using the inappropriate antibiotics to treat an uncertain disease. This intention can be effective thanks to trust in healthcare professionals, and positive experiences with prescribed treatments as well.

*“Excessive consumption… I do not agree… if it is excessive*,* it can become a problem.” (Woman*,* 27 years old*,* Mahajanga I)*.

*“The main reason I am afraid is that the disease is not the same… so I fear there may be consequences later.” (Woman*,* 36 years old*,* Mahajanga II)*.

Among the urban participants, the intention to follow medical advice was often grounded in institutional trust rather than personal conviction, reflecting confidence in the authority of the healthcare system.

*“You can take [antibiotics] if they are prescribed… if it is a health worker who prescribes them*,* you can take them… he gives you the dosage — morning*,* noon*,* and evening — and you follow it properly.” (Mahajanga I*,* 30 years)*.

In contrast, some rural women’s discourse revealed a strong sense of responsibility and caution, emphasizing the protection of their own health and that of their children. For instance, a participant stated, *“Pregnant and breastfeeding women should not take medicines unless they come from the doctor.” (Mahajanga II*,* 27 years)*.

However, this intention could be weakened by distrust or negative medical experiences, as well as by social and structural barriers, including family influence, limited access to healthcare services, or unclear medical prescriptions.

*“Yes! When in doubt*,* I ask health workers for advice*,* they are the doctors.”* (Mahajanga II, 36 years).

### Women’s attitudes toward medical advice

Women expressed a range of beliefs regarding the benefits and risks of adhering to medical prescriptions before using antibiotics. A positive attitude often stemmed from trust in healthcare professionals and the wish to avoid health issues for themselves and their children.

“*I do not take them at all if I don’t understand. If there is no clear prescription*,* I prefer to stop.*” (28 years, Mahajanga II).

“*Even if I have taken that medicine before*,* I go to the health center to get medical advice.*” (26 years, Mahajanga I).

Urban women appeared less represented among those with favorable attitudes, and their narratives were more heterogeneous and sometimes ambivalent, reflecting multiple influences on decision-making, including peers, medical staff, and family members.

“*Sometimes I wait for what my friend tells me to take; if it works for her*,* I also try.*” (Mahajanga I, 25 years).

In rural areas, women’s discourse more frequently reflected awareness of the risks of self-medication during pregnancy and emphasized the protective role of medical supervision. “*Even taking a very small thing can lead to miscarriage if it is not prescribed by a doctor.*” (Mahajanga II, 28 years).

However, some participants reported difficulties in following prescriptions due to unclear medical instructions or concerns about side effects, leading to a form of selective trust toward medical advice.

*“The plants! Antibiotics do not cure me”.* (Mahajanga I, 30 years)

*“No*,* I do not agree with that… if you take too many antibiotics*,* it can cause the baby to be lost instead of helping it grow… they must be taken in moderation.”* (24 years old, Mahajanga II).

### Subjective norms reflecting social influences on medical decisions

Social influences, especially from husbands, mothers-in-law and/or neighbors, were frequently reported as shaping women’s decisions regarding antibiotic use. These people often provided treatment advice that was sometimes aligned with medical recommendations but also promoted informal or nonprescribed practices.

“It’s my mother-in-law who tells me what to buy. She knows the medicines better than I do.” (30 years, Mahajanga I)

“If my husband says the medicine is good, I buy it, even without seeing a doctor.” (27 years, Mahajanga I)

In urban areas, women’s reasoning was more often framed around competence and institutional accountability than communal trust.

*“If the person selling the drug is not well trained*,* I prefer not to buy it.”* (Mahajanga I, 35 years).

In rural areas, restrictive norms were commonly expressed, emphasizing obedience to medical authority and collective disapproval of self-medication. In this sense, rural norms appeared to act as protective mechanisms against inappropriate antibiotic use.

*“Only qualified health workers know which medicine is appropriate.”* (Mahajanga II, 32 years).

Conversely, non-restrictive norms were also observed among some rural participants, who described a widespread tolerance of informal antibiotic practices within their communities.

*“Some take one*,* two*,* or three [tablets]*,* and that’s it; they don’t finish the treatment.”* (Mahajanga II, 27 years).

### Perceived behavioral control: constraints and capacities to act

Women frequently reported various barriers affecting their ability to follow medical advice, including limited access to healthcare services, difficulty understanding prescriptions, and restricted autonomy in making medical decisions. These constraints were often compounded by poor communication and mistrust toward healthcare providers, ultimately shaping their perceived behavioral control over the ability to follow medical advice.

“When I cannot read the prescription, I do not take the medicine. I would rather do nothing.”(28 years, Mahajanga II).

“I only go to the clinic if I am not healed by what I’ve already taken.”(30 years, Mahajanga II).

Urban participants were less represented among women showing strong perceived control. Their accounts rarely highlighted self-regulation, revealing a tendency to delegate decision-making to healthcare providers or pharmacists.

“*If I’m sick*,* I just go to the pharmacy and buy what they give me*.” (Mahajanga I, 27 years).

By contrast, some rural women demonstrated a greater sense of personal responsibility and self-efficacy, often linked to the habit of seeking clarification or repeated consultation.

“*I prefer to go back to the doctor so that he can explain again.*” (Mahajanga II, 29 years).

Nevertheless, limited perceived control emerged in both urban and rural areas, though the underlying factors differed. Among rural women, these barriers were largely practical or cognitive, such as illiteracy, misunderstanding of prescriptions, and lack of decision-making autonomy in healthcare decisions.

“*If I feel the same symptoms*,* I just take the same antibiotic again*” (Mahajanga I, 26 years). This illustrates how familiarity with pharmacies and prior self-medication may foster a false sense of mastery rather than genuine behavioral control.

As illustrated, pregnant and breastfeeding women’s intention to follow medical advice was strengthened by positive attitudes, supportive networks, and healthcare access. Good communication and trust between the prescriber or the doctor and the patient play then a leading part in a positive perceived behavioral control.

### Quantitative analysis of women’s intention to follow medical advice

In urban areas, the mean scores ranged from 4.219 for ATB to 5.312 for the BI (medians 4.250–5.400). SN and PBC averaged 4.625 and 5.176 (medians 4.625 and 5.182).

In rural areas, mean scores ranged from 4.207 for ATB to 5.179 for BI (medians 4.167–5.400). SN and PBC averaged 4.446 and 5.041 (medians 4.562 and 5.045). Overall, mean scores were slightly higher in urban areas across all constructs, indicating marginally more favorable attitudes and intentions among urban respondents.

Linear regression analyses revealed that in urban areas, SN were the strongest predictor, whereas in rural areas, PBC was the main predictor of behavioral intention.

### Subjective norms as the primary predictor of TPB constructs in urban areas

**Fig. 4:**
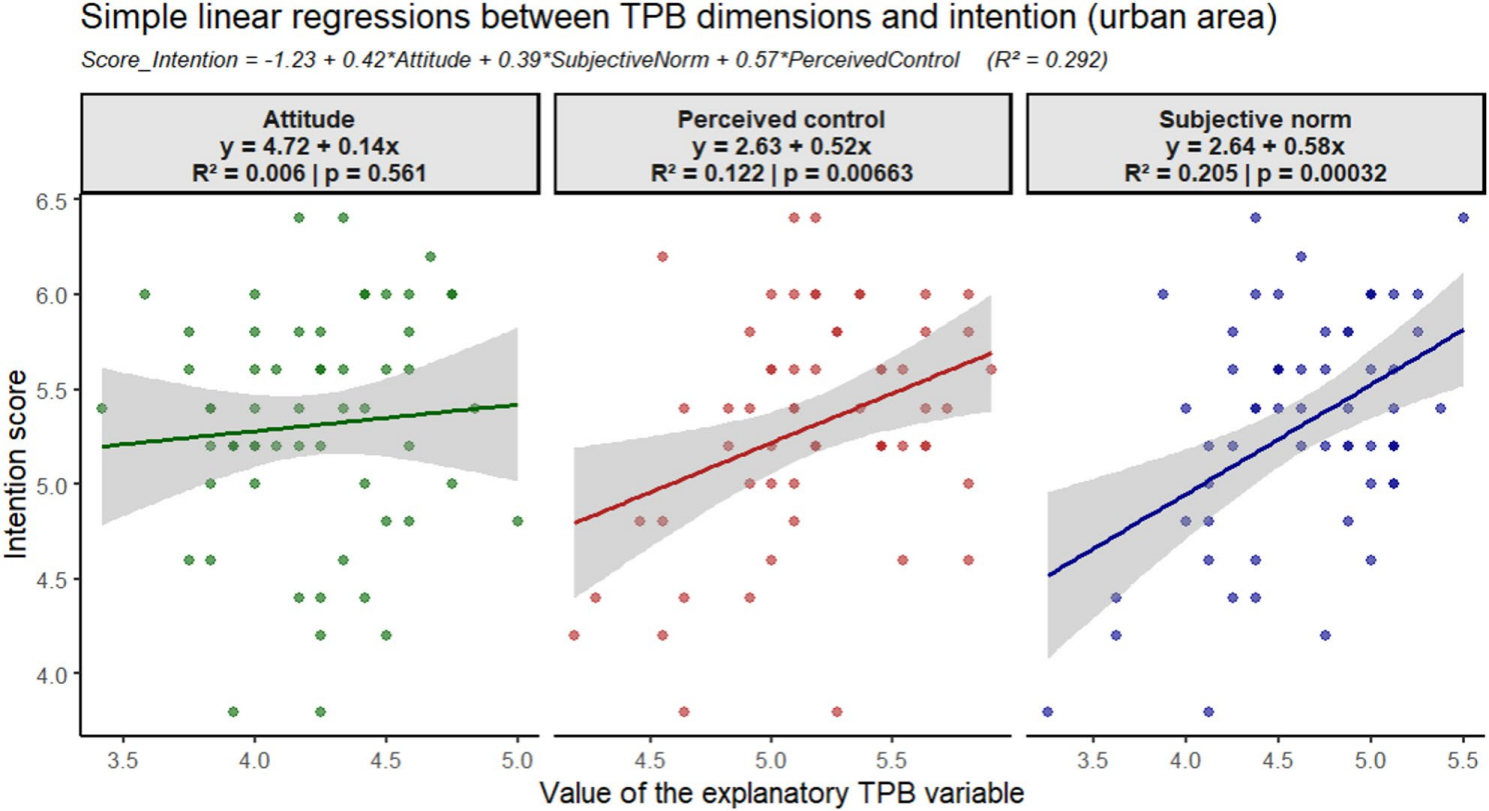
Simple linear regressions of the intention score and TPB constructs in urban areas

In urban areas, simple linear regression between each construct score of the TPB and the BI scores revealed that SN were the strongest explanatory factor, with a slope of 0.58 and a coefficient of determination of *R²* = 0.205 (*p* = 0.00032) [Fig. 4]. This association indicates that the more women perceive social pressure or normative expectations to engage in supervised antibiotic use, the stronger their intention to follow medical advice.

PBC showed a positive but moderate association with BI, explaining 12.20% of its variance (R² = 0.122, *p* = 0.00663). This suggests that women with greater access to care, confidence, or resources expressed stronger intentions to comply, which highlights the importance of perceived ability and support in translating intention into action.

In the multiple linear regression model including all three TPB constructs, a one-point increase in ATB, SN, and PBC corresponded to 0.42-, 0.39-, and 0.57-point increases in BI, respectively, after controlling for other variables. The model accounted for 29.20% of the variance in women’s BI (R² = 0.292), reflecting a moderate yet meaningful explanatory power of the TPB in urban contexts.

The equation for the overall multiple regression model, displayed at the top of the figure, is as follows: BI score = – 1.23 + 0.42 ×ATB + 0.39 × SN + 0.57 × PBC [Fig. 4].

### Perceived behavioral control as the primary predictor of the TPB construct for intention in rural areas

In rural areas, simple regression revealed that PBC was the strongest predictor of intention (β = 0.58, R² = 0.214, *p* = 0.000337), explaining 21.4% of its variance. This highlights that women’s confidence in their ability through access, knowledge, or resources significantly strengthened their intention to follow medical advice on antibiotic use.

**Fig. 5:**
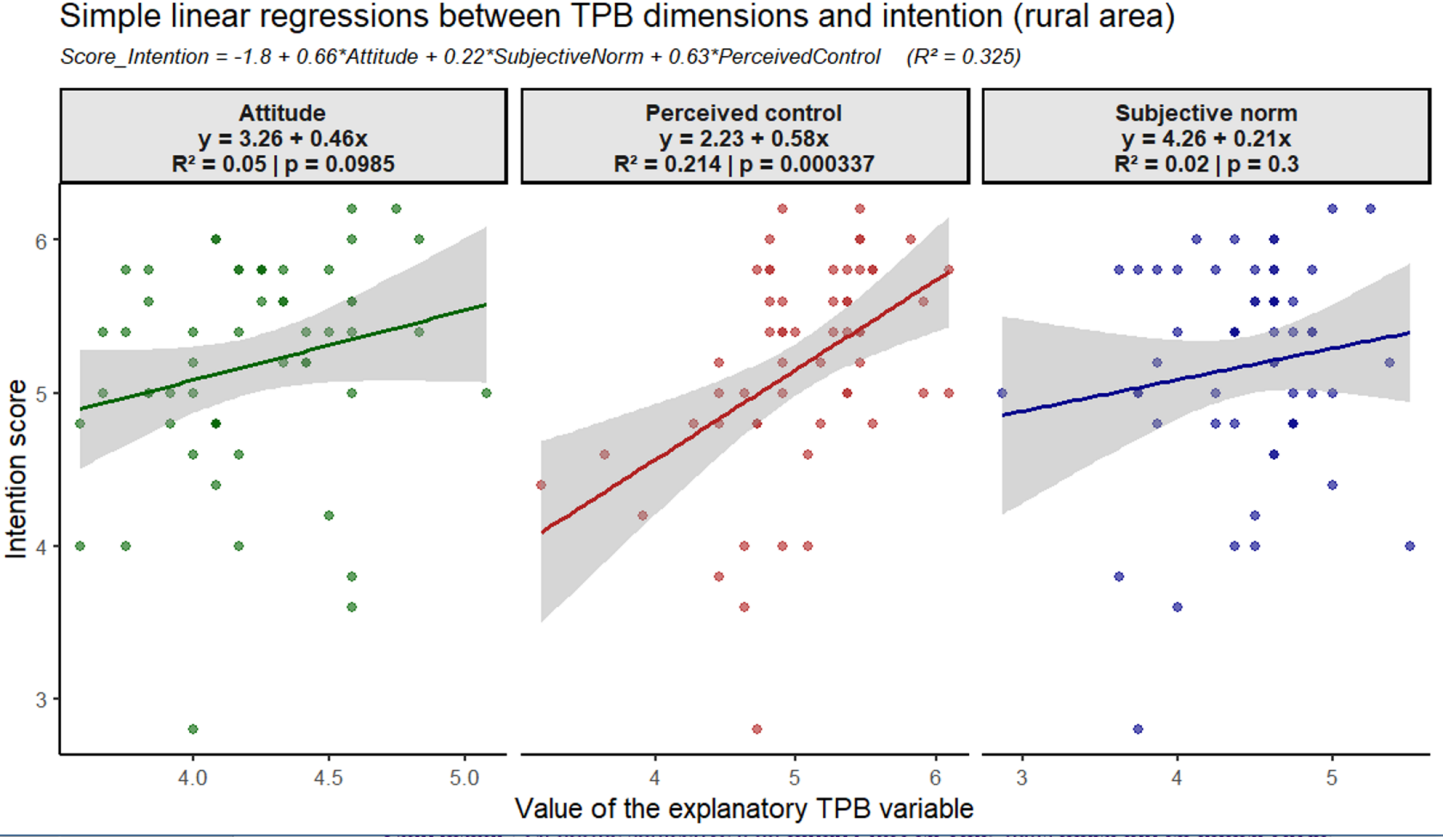
Simple linear regressions of the intention score and TPB constructs in rural areas

ATB were positively but weakly associated with BI (R² = 0.050, *p* = 0.0985) [Fig. 5]. Although not statistically significant, this trend suggests that favorable views of rational antibiotic use may increase BI, but ATB alone was not a robust predictor in the univariate model.

In rural areas, the multiple linear regression model [Fig. 5] explained 32.5% of the variance in BI (R² = 0.325), which was slightly greater than that in urban areas. A one-point increase in ATB corresponded to a 0.66-point rise in BI, SN to a 0.22-point rise, and PBC to a strong 0.63-point rise, nearly equal to ATB. These results indicate that the TPB has moderate explanatory power in rural contexts, with PBC emerging as the dominant determinant of BI.

The equation for the overall multiple regression model, displayed at the top of the figure, is as follows:

BI score = − 1.8 + 0.66 ×ATB + 0.22 × SN + 0.63 × PBC [Fig. 5].

### Comparison of linear regression results between urban and rural areas

In urban areas, SN exerted the strongest influence, whereas in rural contexts, their effect was not significant. PBC was important in both areas but more decisive in rural areas, reflecting greater resource constraints. ATB also played a stronger role in rural contexts than in urban contexts. Multiple regression confirmed the relevance of the TPB across environments, although with different weightings: In urban areas BI was driven mainly by SN, followed by PBC and ATB, whereas in rural areas BI was dominated by PBC, with ATB and SN as secondary predictors.

### Description of intention levels to follow medical advice prior to antibiotic use in urban and rural areas

More than half (56.52%) of the women expressed unfavorable attitudes toward medical advice, especially among those with low BI, suggesting that attitudes may hinder adherence. Perceived SN were evenly split into 50.43% perceived nonrestrictive (or weak) social pressure vs. 49.57% perceived restrictive (or strong) social pressure, reflecting diverse social influences without a dominant trend. With respect to PBC, a slight majority (53.91%) reported limited PBC, suggesting that reduced personal agency may act as a barrier, particularly for women with lower intentions [Table 2].

**Table 2:** Level of women’s intention to follow medical advice according to psychosocial and sociodemographic determinants

Variables / Areas	Urban areas				Rural areas			
	*p*-value	Low intention *N* (%)	High intention *N* (%)		*p*-value	Low intention *N* (%)	High intention *N* (%)	Total *N* (%)
Attitudes toward medical advice								
Unfavorable	0.3486	23(38.98)		12(20.34)	**0.0379**	23(41.07)	7(12.50)	65(56.52)
Favorable		12(20.34)		12(20.34)		12(21.43)	14(25.00)	50(43.48)
Perceived subjective norms								
Non-restrictive	0.7092	19(32.20)		11(18.64)	1	18(32.14)	10(17.86)	58(50.43)
Restrictive		16(27.12)		13(22.03)		17(30.36)	11(19.64)	57(49.57)
Perceived behavioral control								
Limited	0.4755	22(37.29)		12(20.34)	**0.0977**	21(37.50)	7(12.50)	62(53.91)
Important		13(22.03)		12(20.34)		14(25.00)	14(25.00)	53(46.09)
Antibiotic consumption period of use								
1 month or less	0.3665	15(25.42)		14(23.73)	1	11(19.64)	6(10.71)	46(40.00)
2–3 months		20(33.90)		10(16.95)		24(42.86)	15(26.79)	69(60.00)
Place of antibiotic used acquisition								
Non official	0.294	1(1.69)		3(5.08)	**0.0188**	2(3.57)	7(12.50)	13(11.30)
Official		34(57.63)		21(35.59)		33(58.93)	14(25.00)	102(88.70)
Person who recommended antibiotics used								
Health Worker	1	27(45.76)		18(30.51)	0.5888	30(53.57)	16(28.57)	91(79.13)
Other people		8(13.56)		6(10.17)		5(8.93)	5(8.93)	24(20.87)
Woman status								
Breastfeeding	0.3882	33(55.93)		21(35.59)	0.4586	28(50)	19(33.93)	101(87.83)
Pregnant		2(3.39)		3(5.08)		7(12.50)	2(3.57)	14(12.17)
Gravidity								
2 or less	1	20(33.9)		14(23.73)	1	19(33.93)	11(19.64)	64(55.65)
3 or more		15(25.42)		10(16.95)		16(28.57)	10(17.86)	51(44.35)
Age category								
≥ 27 Years	1	23(38.98)		15(25.42)	1	13(23.21)	8(14.29)	59(51.30)
< 27 Years		12(20.34)		9(15.25)		22(39.29)	13(23.21)	56(48.70)
Marital status								
Married	1	32(54.24)		22(37.29)	**0.1785**	25(44.64)	19(33.93)	98(85.22)
Non married		3(5.08)		2(3.39)		10(17.86)	2(3.57)	17(14.78)
Employment								
Other	0.8412	7(11.86)		4(6.78)	**0.0598**	16(28.57)	15(26.79)	42(36.52)
Trader		16(27.12)		10(16.95)		6(10.71)	4(7.14)	36(31.30)
Housewife		12(20.34)		10(16.95)		13(23.21)	2(3.57)	37(32.17)
Educational level								
≥ Secondary	0.3853	30(50.85)		23(38.98)	**0.0514**	22(39.29)	19(33.93)	94(81.74)
< Secondary		5(8.47)		1(1.69)		13(23.21)	2(3.57)	21(18.26)

In urban areas, bivariate analysis revealed no significant associations between BI and psychosocial or sociodemographic variables, including SN (*p* = 0.7092), ATB (*p* = 0.3486), and PBC (*p* = 0.4755). However, low-intention women were more often characterized by unfavorable ATB (38.98%) and limited PBC (37.29%).

In rural areas, bivariate analyses revealed that unfavorable ATB were more common among women with low BI (41.07%; *p* = 0.0379), as was limited PBC (37.50%; *p* = 0.0977). Surprisingly, women with low BI more often obtained antibiotics through official sources (58.93%; *p* = 0.0188). Higher education was positively linked to stronger BI (33.93% vs. 3.57%; *p* = 0.0514), whereas being a housewife was more frequent among women with low BI (23.21%; *p* = 0.0598). These findings suggest that ATB, PBC, education, and occupation may influence adherence to medical advice in rural areas [Table 2].

### Predictors of women’s intention to follow medical advice

In urban areas, the multivariable logistic regression revealed that favorable ATB were marginally associated with stronger BI (aOR = 3.69, *p* = 0.056). High PBC also showed a borderline association (aOR = 3.12, *p* = 0.094). Conversely, obtaining antibiotics from official sources showed a nonsignificant trend toward a negative association with strong BI (aOR = 0.16, *p* = 0.142) [Table 3].

**Table 3:** Predictors of the intention to follow medical advice in urban areas

Variables	Estimate	Std. Error	z value	*p*-value	aOR	CI 95%
Attitudes toward the behavior (ATB)						
Unfavorable	Ref.					
Favorable	1.3064	0.6848	1.908	**0.056**	3.69	[1.03–15.97]
Perceived behavioral control (PBC)						
Limited	Ref.					
Important	1.1381	0.6793	1.675	**0.094**	3.12	[0.88–13.31]
Place of antibiotic used acquisition						
Non official	Ref.					
Official	-1.8190	1.2389	-1.468	0.142	0.16	[0.007–1.48]

Statistically, the model showed a modest fit (AIC = 80.73). Although the associations did not reach significance, ATB and PBC appeared to influence BI.

In rural areas, the multivariable logistic regression model revealed that a favorable ATB was strongly associated with higher BI. Women with favorable ATB were up to 225 times more likely to intend to follow medical advice (*p* = 0.005). PBC was also a significant predictor: compared with women reporting low PBC, those with high PBC were 24.7 times more likely to express a strong intention to follow medical recommendations (*p* = 0.029).

Obtaining medications from official sources (pharmacy, health center) was associated with lower BI. Women who acquired antibiotics through official channels had a 99% lower likelihood (*p* = 0.037) of seeking medical advice before using them. This unexpected finding warrants further investigation into distribution practices, prescription procedures, and possible self-medication behaviors.

In addition, sociodemographic factors, including being unmarried, having three or more pregnancies, and being a housewife, were also significantly associated with reduced the BI. Women with three or more pregnancies had a 94% lower probability of intending to follow medical advice than those with two or fewer pregnancies did (*p* = 0.020). Unmarried women had a 99% lower probability of high BI than married women did (*p* = 0.030). Housewives were also less likely to intend to follow medical recommendations, with a 90% reduction compared with women who engaged in other activities (*p* = 0.006) [Table 4].

**Table 4:** Predictors of the intention to follow medical advice in rural areas

Variable	Estimate	Std. Error	z value	*p*-value	aOR	CI 95%
Attitudes toward the behavior (ATB)						
Unfavorable	Ref.					
Favorable	5.417	1.943	2.788	**0.005**	225.10	[10.48–24739.82]
Subjective norms (SN)						
Non-restrictive	Ref.					
Restrictive	1.498	1.046	1.432	0.152	4.47	[0.68–47.89]
Perceived behavioral control (PBC)						
Limited	Ref.					
Important	3.190	1.459	2.186	**0.029**	24.27	[2.25–849.86]
Place of antibiotic used acquisition						
Non official	Ref.					
Official	-6.556	3.142	-2.086	**0.037**	0.001	[0.000001–0.19]
Gravidity						
2 or less	Ref.					
3 or more	-2.892	1.241	-2.329	**0.020**	0.06	[0.003–0.46]
Marital status						
Married	Ref.					
Non married	-5.616	2.582	-2.175	**0.030**	0.001	[0.000005–0.18]
Employment						
Other	Ref.					
Trader	-1.537	1.288	-1.193	0.233	0.21	[0.01–2.20]
Housewife	-4.654	1.696	-2.745	**0.006**	0.01	[0.0001–0.15]

The model showed a good fit (AIC = 51.56), confirming the relevance of the TPB in rural contexts while highlighting social vulnerabilities and structural barriers that may limit women’s capacity or willingness to follow medical advice.

### Comparative analysis of urban and rural contexts

The comparative analysis revealed marked urban–rural contrasts in the predictors of women’s intention to follow medical advice. Across both areas, the TPB constructs, particularly ATB and PBC, were relevant, but their strength differed. In urban areas, the associations were weak and nonsignificant, whereas in rural areas, they were strong and statistically robust, indicating a greater influence of psychosocial factors.

A negative association between BI and obtaining medication from official sources was observed in both urban and rural contexts but reached statistical significance only in rural areas, which suggests possible issues of trust or access in formal healthcare. The rural model also revealed significant sociodemographic predictors, such as being unmarried, having high parity, and lacking employment, each linked to reduced BI, whereas no such effects appeared in the urban model.

Model fit was stronger in rural areas (AIC = 51.56 vs. 80.73), underscoring the robustness of these findings.

## Discussion

This study investigated the cognitive and psychosocial predictors of pregnant and breastfeeding women’s intention to follow medical advice before antibiotic use and compared urban and rural contexts in Madagascar. Guided by the theory of planned behavior (TPB) and a mixed-methods design, the analysis revealed the following area-specific patterns: subjective norms (SN) were most influential in urban areas, whereas perceived behavioral control (PBC) and attitude toward the behavior (ATB) were decisive in rural areas.

### Methodological considerations

This exploratory study has certain limitations, including the small number of women per site and the use of convenience sampling, which constrain the robustness of the quantitative analyses and limit both extrapolation to other regions and the statistical representativeness of the findings at the national level. In addition, the qualitative grouping of items within each TPB construct, despite the precautions taken by our team, could be interpreted differently by other researchers. Robustness was strengthened through the use of multiple regression, PLS-R, and binary logistic regression, mitigating the limitations of any single method.

### Patterns of antibiotic use

Compared with rural women, urban women reported more recent antibiotic use, likely reflecting greater access to pharmacies and clinics. Similar patterns have been observed in East Africa, where urban residents are more prone to self-medication [32, 33]. In both areas, 21% of women obtained antibiotics from nonmedical sources, echoing evidence from other African countries that informal markets remain a significant supply channel [34, 35].

In rural areas, being unmarried, multiparous, or a housewife was linked to lower intention, reflecting reduced autonomy, financial dependence, and time constraints; these patterns are consistent with those of other African studies [36]. These associations were not observed in urban areas, where broader access to education and services may offset social and economic vulnerabilities.

### Determinants of the intention to follow medical advice

In urban areas, SN consistently emerged as the strongest predictor of intention across statistical approaches (MLR and PLS-R), highlighting the influence of family, peers, and healthcare providers in shaping women’s decisions. These findings are consistent with those of studies in Nigeria and Vietnam, where social expectations reinforced adherence to medical advice [37, 38]. PBC also contributed, highlighting that even when intention is present, the ability to act through literacy, access, or confidence remains critical.

In rural areas, PBC was the dominant predictor across the models (MLR, PLS-R, and BLR), highlighting how structural barriers such as distance, transport costs, and literacy limit women’s ability to follow medical advice. Similar constraints have been reported in Uganda and Tanzania [39, 40]. ATB also significantly influenced BI, underscoring the importance of favorable beliefs about consultation, suggesting that decision-making is shaped more by practical realities than by social pressure. Unexpectedly, women who obtained antibiotics from official sources were less likely to report strong BI. These counterintuitive results warrant further study of distribution practices, prescription procedures, and self-medication and may reflect mistrust, stockouts, or poor provider communication.

### Qualitative insights and study limitations

Qualitative narratives enriched the quantitative results by clarifying how women interpreted medical advice and navigated treatment decisions. Unclear instructions led women to stop treatment, highlighting the need for clearer communication [41]. Family members, particularly husbands and mothers-in-law, could either reinforce or contradict medical advice, which is consistent with evidence that family dynamics strongly shape health behaviors [42, 43]. Limited literacy and financial barriers further highlight the role of PBC, echoing findings from other African contexts where structural barriers constrain access to care [44, 45]. BI was strong when women trusted providers and recognized the risks of self-medication, which is consistent with research linking trust in healthcare professionals to better adherence [46]. These insights affirm the relevance of the TPB while showing how structural and relational factors shape health behaviors.

A key strength of this study is its mixed-methods design and triangulation of analytical approaches, which enhanced quantitative and qualitative validity and allowed comparisons across urban and rural contexts [47]. Limitations include the small sample size, and the reliance on self-reported data, increasing the possibility of recall and social desirability bias. The binary logistic regression results should be interpreted cautiously given the wide confidence intervals. Nonetheless, convergence across methods and qualitative validation support confidence in the core findings.

### Public health implications and recommendations

These findings highlight the need for context-specific strategies to strengthen women’s intention to follow medical advice. In urban areas, BI should be focused on reinforcing positive SN and trust among healthcare providers. Public health campaigns could engage community leaders, traditional birth attendants, and pharmacists to promote consultation with professionals while improving prescription clarity and provider–patient communication. In rural areas, priorities include expanding access through mobile clinics, reducing consultation costs, and enhancing women’s literacy and autonomy. Economic and social empowerment initiatives may further strengthen PBC and ATB toward medical advice. Cross-cutting strategies should engage community health workers and local leaders as trusted intermediaries to promote rational antibiotic use. Prescription-only regulations need to be paired with educational campaigns adapted to local languages and literacy levels.

## Conclusion

This study demonstrates the contextual relevance of the theory of planned behavior (TPB) in explaining women’s intention to follow medical advice before antibiotic use in Madagascar. SN were central in urban areas, whereas PBC and ATB dominated in rural contexts. Structural barriers, mistrust of formal health services, and social vulnerabilities further constrained BI, particularly in rural areas.

By triangulating multiple analytical approaches (MLR, PLS-R, BLR) and integrating qualitative interviews, we strengthened the robustness and interpretation of the findings. Despite the study limitations, the theoretical alignment and mixed-methods design support the validity of the results.

Our findings highlight the need for context-specific interventions. In urban areas, strategies should strengthen trust in providers, reinforce positive social norms, and improve the clarity of prescriptions through peer educators, pharmacists, and community leaders. In rural areas, improving structural access—via mobile clinics, subsidized consultations, and community-based education—remains essential, alongside enhancing women’s autonomy, literacy, and financial independence.

Cross-cutting strategies should mobilize community health workers as trusted intermediaries and combine restrictions on nonprescription sales with culturally tailored educational campaigns. Ultimately, promoting rational antibiotic use among pregnant and breastfeeding women requires systemic changes that address communication gaps, reduce access barriers, and strengthen social support. Tailored approaches adapted to urban and rural realities are critical for safeguarding maternal and child health and contributing to global efforts against antimicrobial resistance.

## Supplementary Information

Supplementary Material 1.

Supplementary Material 2.

Supplementary Material 3.

## Data Availability

The datasets generated during and/or analysed during the current study are available from the corresponding author on reasonable request. Some data and materials are also provided within the manuscript and in the supplementary information file (Supplementary Table S1).

## Abbreviations

AMR: Antimicrobial Resistance
ATB: Attitude toward the behavior
BLR: Binary logistic regression
MLR: Multiple Linear Regression
SN: Subjective norms
PBC: Perceived behavioral control
PLS-R: Partial Least Squares Regression
TPB: Theory of Planned Behavior
WHO: World Health Organization
