# Supplementary material for: Pregnant and breastfeeding women’s intention to follow medical advice before antibiotic use: a comparative pilot analysis using the theory of planned behavior in Mahajanga, Madagascar
Source: BMC Public Health. 2026 Jan 12;26:521. doi: 10.1186/s12889-026-26190-1 (PMC12888152; doi:10.1186/s12889-026-26190-1)
Supplement: Supplementary file 1 — Supplementary Material 1. [file 12889_2026_26190_MOESM1_ESM.docx]

**Supplementary Table S1:** Items with their main theme, polarity, associated theory of planned behavior constructs, and illustrative quotes

| **N** | **Statement (items)** | **Main theme** | **Expected polarity** | **Theory of planned behavior constructs** | **llustrative quotes** |
| --- | --- | --- | --- | --- | --- |
| 1 | The consumption of antibiotics causes diseases in mothers and children. | Perception of antibiotics | Negative | Attitude toward the behavior | *“The main reason I am afraid is that the illness is not the same… so I fear there may be consequences later.” (Woman, 36 years old, Mahajanga II)* |
| 2 | Excessive consumption of an antibiotic can make it even more effective. | Perception of antibiotics | Negative | Attitude toward the behavior | *“Excessive consumption… I do not agree… if it is excessive, it can become a problem.” (Woman, 27 years old, Mahajanga I)* |
| 3 | For any illness in pregnant women, antibiotics should be taken. | Perception of antibiotics | Negative | Attitude toward the behavior | *“After childbirth… it is necessary to take antibiotics prescribed by the doctor.” (Woman, 29 years old, Mahajanga II)* |
| 4 | Pregnant and breastfeeding women can take antibiotics in their own way (dose, duration, frequency). | Perception of medication consumption | Negative | Perceived behavioral control | *“It is not good to take too much… it has already affected my stomach.” (Woman, 22 years old, Mahajanga II)* |
| 5 | Healthcare providers always communicate the possible effects of antibiotics on the health of the mother and child. | Communication between the medication prescriber and the patient | Positive | Subjective norms | *“It is better to go to a health center… you will receive instructions on the correct dosage of the medicines.” (Woman, 25 years old, Mahajanga II)* |
| 6 | If I take antibiotics before my delivery, I inform the doctor. | Communication between the medication prescriber and the patient | Positive | Behavioral Behavioral intention | *“To be honest, pregnant and breastfeeding women should not take medicines unless they come from the doctor.” (Woman, 27 years old, Mahajanga II)* |
| 7 | If I take antibiotics without a doctor's advice during my pregnancy, my health and that of the newborn should be monitored. | Perception of medication prescriptions | Positive | Behavioral intention | *“Pregnant and breastfeeding women should not take medicines without the doctor, because there are consequences for both the baby and the mother.” (Woman, 31 years old, Mahajanga II)* |
| 8 | Excessive antibiotic use during pregnancy promotes fetal growth. | Perception of antibiotics | Negative | Attitude toward the behavior | *“No, I do not agree with that… if you take too many antibiotics, it can cause the baby to be lost instead of helping it grow… they must be taken in moderation.” (Woman, 24 years old, Mahajanga II)* |
| 9 | Healthcare providers prescribe too many antibiotics to pregnant and breastfeeding women. | Perception of medication prescriptions | Negative | Subjective norms | *“The doctor told me that I had to take medicines to avoid getting sick, reduce my pain, and that it was necessary after childbirth.” (Woman, 35 years old, Mahajanga II)* |
| 10 | Antibiotics are overconsumed by the general public. | Perception of community consumption | Negative | Subjective norms | *“Most people practice self-medication because life is difficult.” (Woman, 26 years old, Mahajanga II)* |
| 11 | I can use an antibiotic without reading or having someone read its leaflet for me. | Perception of medication consumption | Negative | Perceived behavioral control | *“I do not understand what is written… I always take amoxicillin when I am in pain.” (Woman, 20 years old, Mahajanga II)* |
| 12 | If in doubt, I seek the advice of a health worker before taking an antibiotic. | Communication between the medication prescriber and the patient | Positive | Behavioral intention | *“Yes! When in doubt, I ask health workers for advice, they are the doctors.” (Woman, 36 years old, Mahajanga II)* |
| 13 | I report any side effects caused by taking antibiotics to other people (family, friends). | Community communication | Positive | Subjective norms | *“I always pay attention to my health… but I have not yet experienced any strange reactions.” (Woman, 22 years old, Mahajanga II)* |
| 14 | Combining antibiotics with traditional medicines can speed up disease recovery. | Perception of alternatives | Negative | Attitude toward the behavior | *“I also use plants… but if the doctor prescribes, I prefer to follow that.” (Woman, 28 years old, Mahajanga II)* |
| 15 | Taking antibiotics during pregnancy prevents miscarriage. | Perception of antibiotics | Negative | Attitude toward the behavior | *“I did not agree, because a pregnant woman is fragile. Even taking a very small thing can lead to miscarriage if it is not prescribed by a doctor.” (Woman, 33 years old, Mahajanga II)* |
| 16 | If a doctor prescribes medication, I can take them all without following the prescription. | Perception of medication consumption | Negative | Perceived behavioral control | *“One cannot accept that! Because you must follow the doctor’s prescription when taking medicines. Otherwise, the dose may be excessive or insufficient.” (Woman, 24 years old, Mahajanga II)* |
| 17 | Antibiotics are more effective than other medicines. | Perception of antibiotics | Negative | Attitude toward the behavior | *“There are other medicines that are more effective… the plants! Antibiotics do not cure me. I have already used them but they have never been effective for me, whereas plants have.” (Woman, 32 years old, Mahajanga I)* |
| 18 | Medicines can be purchased anywhere. | Perception of community consumption | Negative | Perceived behavioral control | *“I do not agree! Like in grocery stores… there are things you do not know, and you need to ask. But they cannot explain, unlike qualified pharmacists. I never go to grocery stores, always to the pharmacy.” (Woman, 36 years old, Mahajanga II)* |
| 19 | After childbirth, antibiotics should be systematically given to women. | Perception of medication prescriptions | Negative | Attitude toward the behavior | *“I agreed because they must heal the wound in the abdomen… after childbirth, you need to take them.” (Woman, 30 years old, Mahajanga II)* |
| 20 | The duration of antibiotic use depends on the progress of disease recovery. | Perception of medication consumption | Negative | Attitude toward the behavior | *“I agreed because there are different types of illnesses, so the antibiotics taken must also change. […] When I take them and see that they make me healthy, I continue.” (Woman, 36 years old, Mahajanga II)* |
| 21 | When the prescription is not readable, I take the medicine in my own way. | Perception of medication consumption | Negative | Perceived behavioral control | *“Since I do not understand the doctor’s handwriting, I prefer to go back to him so that he can explain, in order to follow the instructions.” (Woman, 20 years old, Mahajanga II)* |
| 22 | A pregnant or breastfeeding woman can take multiple antibiotics prescribed by a healthcare provider. | Perception of medication prescriptions | Positive | Subjective norms | *“You can take them if they are prescribed… if it is a health worker who prescribes them, you can take them… but only when… you go to him, say you are pregnant, and he gives you the antibiotic with the dosage—morning, noon, and evening—and you follow it properly.” (Woman, 30 years old, Mahajanga I)* |
| 23 | The treatment of pregnant women with antibiotics should be monitored by prescribers. | Perception of medication prescriptions | Positive | Behavioral intention | *“This requires monitoring, because without follow-up and in case of error with the medicines, it can make you sick.” (Woman, 22 years old, Mahajanga II)* |
| 24 | All medicine sellers should be trained on antibiotic prescriptions. | Perception of medication prescriptions | Positive | Subjective norms | *“When they sell medicines, they should ask where the person is hurting… and provide the right medicine. All sellers should be trained.” (Woman, 30 years old, Mahajanga I)* |
| 25 | Drinking plenty of water helps reduce the possible negative effects of antibiotics. | Perception of medication consumption | Positive | Attitude toward the behavior | *“That is true, you really need to drink plenty of water so that the antibiotic goes down, the medicine you take. […] It is good for your health; drinking a lot of water is also good for your health.” (Woman, 26 years old, Mahajanga II)* |
| 26 | Only qualified healthcare personnel should prescribe antibiotics to pregnant and breastfeeding women. | Perception of medication prescriptions | Positive | Subjective norms | *“Yes, I agreed because only qualified health workers know which medicine is appropriate… You should never buy in grocery stores or from people passing by. Medicines must come from the pharmacy.” (Woman, 24 years old, Mahajanga II)* |
| 27 | Antibiotics can always be requested during a consultation. | Perception of medication prescriptions | Negative | Perceived behavioral control | *“Yes, you can! For our health, we always need that!” (Woman, 32 years old, Mahajanga I)* |
| 28 | Always follow the advice of your health professional when using antibiotics. | Communication between the medication prescriber and the patient | Positive | Behavioral intention | *“I agreed because… it makes you healthy, I think it is good.” (Woman, 34 years old, Mahajanga II)* |
| 29 | I can share my antibiotics with other people. | Perception of community consumption | Negative | Perceived behavioral control | *“I cannot share them, the illness is not the same… they must go to the doctor when they are sick.” (Woman, 36 years old, Mahajanga II)* |
| 30 | I can use leftover antibiotics from a previous treatment if the symptoms seem the same. | Perception of medication consumption | Negative | Perceived behavioral control | *“When the symptoms are the same… I take the leftover amoxicillin… but if it is not the same illness, I go to the doctor.” (Woman, 19 years old, Mahajanga II)* |
| 31 | Communities know how to use antibiotics properly. | Perception of community consumption | Negative | Subjective norms | *“Sometimes it does not conform to the instructions of community health workers… some take one, two, or three…” (Woman, 21 years old, Mahajanga II)* |
| 32 | Traditional medicines (leaves and herbs) can be used instead of antibiotics. | Perception of alternatives | Negative | Attitude toward the behavior | *“For example, with a cough! As soon as I have a sore throat, I go buy mafahy mamy, which replaces antibiotics… We boil it and drink it.” (Woman, 30 years old, Mahajanga I)* |
| 33 | The use of antibiotics in pregnant women should only consider the health of the fetus. | Perception of antibiotics | Negative | Attitude toward the behavior | *“The use of antibiotics in pregnant women should consider only the health of the fetus. Yes, that is absolutely true.” (Woman, 31 years old, Mahajanga I)* |
| 34 | All antibiotics can be prescribed to pregnant women regardless of the stage of pregnancy. | Perception of antibiotics | Negative | Perceived behavioral control | *“I do not agree because a pregnant woman should not take antibiotics! Unless it is prescribed by a health worker, then yes, you can take it. But if, for example, you already have the antibiotic, or if you only buy it at the grocery store, that is not acceptable! Only health workers know that you are pregnant, and they are the ones who know at what stage of pregnancy you are and what you can take.” (Woman, 24 years old, Mahajanga II)* |
| 35 | The dosage must be written in the mother’s health record. | Communication between the medication prescriber and the patient | Positive | Perceived behavioral control | *“I agreed because if it is an overdose, it causes other problems… the doctor monitors which medicines I have already received or not.” (Woman, 22 years old, Mahajanga II)* |
| 36 | I buy antibiotics directly without the advice of a health worker or without a prescription. | Perception of community consumption | Negative | Perceived behavioral control | *“I do not agree because… it is different from having a prescription from the health center! You go to the doctor, and not buy medicines yourself clandestinely.” (Woman, 27 years old, Mahajanga II)* |
