# Supplementary material for: Pregnant and breastfeeding women’s intention to follow medical advice before antibiotic use: a comparative pilot analysis using the theory of planned behavior in Mahajanga, Madagascar
Source: BMC Public Health. 2026 Jan 12;26:521. doi: 10.1186/s12889-026-26190-1 (PMC12888152; doi:10.1186/s12889-026-26190-1)
Supplement: Supplementary file 2 — Supplementary Material 2. [file 12889_2026_26190_MOESM2_ESM.docx]

**Supplementary File (S2):** Study questionnaire and interview guide for pregnant and breastfeeding women in urban and rural areas of Mahajanga, Madagascar

**QUESTIONNAIRE**

**A. Participant eligibility and consent**

| **Variable** | **Response options** |
| --- | --- |
| Is the respondent pregnant and/or breastfeeding? | Pregnant / Breastfeeding / Pregnant & breastfeeding |
| Unique participant ID | Auto‑generated |
| Consent obtained | Yes / No |

**B. Residence information**

| **Variable** | **Response options** |
| --- | --- |
| District | Mahajanga I / Mahajanga II |
| Commune | : ……….. |
| Fokontany | : ……….. |
| Village / Sector | : ……….. |
| GPS coordinates of the interview location | Longitude (X) : ………..  Latitude (Y) : ……….. |

**C. Antibiotic exposure**

| **Variable** | **Response options** |
| --- | --- |
| Taken antibiotics in last 3 months? | Yes / No |
| Taken other medication? | Yes / No |
| Specify other medication | Text |
| Time since last antibiotic use | 1 / 2 / 3 months / Ongoing |
| Source of medication | Pharmacy / Shop / Clinic / Leftover / Friend‑family / Other |
| Who recommended the medication? | Self / Family / Friend / Health worker / Vendor / Other |

**D. Maternal status**

| **Variable** | **Response options** |
| --- | --- |
| Maternal status | Pregnant / Breastfeeding / Both |
| Gestational age (if pregnant) | <3 / 4‑6 / 7‑9 months |
| Gravidity | Number : ……….. |
| Usual antibiotic use in pregnancy | Yes / No / Don’t know |
| Age of breastfeeding child | Years + Months |

**E. Sociodemographic characteristics**

| **Variable** | **Response options** |
| --- | --- |
| Age | In years : ……….. |
| Ethnicity | Sakalava / Antandroy / Betsileo / Merina / Antesaka / Other |
| Marital status | Married / Single / Widow / Cohabitation / Divorced |
| Religion | Catholic / Protestant / Muslim / Other |
| Primary occupation | Livestock farmer / Agricultural worker / Trader / Housewife / Government employee / Other primary occupation (please specify) |
| Secondary occupation | Livestock farmer / Agricultural worker / Trader / Housewife / Government employee / Other primary occupation (please specify) |
| Education level | None / Primary / Secondary / University / Literate |
| Household size | Number: ……….. |
| Total children | Number: ……….. |
| Children <5 years | Number: ……….. |

**F. Antibiotic belief statements (Q‑Sort items)**

We will now ask for your opinion on several statements. Together, we will sort them on a grid according to YOUR point of view. You may read the statements yourself, or we can read them aloud for you. Once you have completed the sorting, we will discuss the statements you strongly agreed with (+3) and the those you strongly disagreed with (–3).

Participants ranked agreement (−3 = strongly disagree to +3 = strongly agree).

| **N** | **Statement (items)** | **Score (−3 to +3)** |
| --- | --- | --- |
| 1 | The consumption of antibiotics causes diseases in mothers and children. |  |
| 2 | Excessive consumption of an antibiotic can make it even more effective. |  |
| 3 | For any illness in pregnant women, antibiotics should be taken. |  |
| 4 | Pregnant and breastfeeding women can take antibiotics in their own way (dose, duration, frequency). |  |
| 5 | Healthcare providers always communicate the possible effects of antibiotics on the health of the mother and child. |  |
| 6 | If I take antibiotics before my delivery, I inform the doctor. |  |
| 7 | If I take antibiotics without a doctor's advice during my pregnancy, my health and that of the newborn should be monitored. |  |
| 8 | Excessive antibiotic use during pregnancy promotes fetal growth. |  |
| 9 | Healthcare providers prescribe too many antibiotics to pregnant and breastfeeding women. |  |
| 10 | Antibiotics are overconsumed by the general public. |  |
| 11 | I can use an antibiotic without reading or having someone read its leaflet for me. |  |
| 12 | If in doubt, I seek the advice of a health worker before taking an antibiotic. |  |
| 13 | I report any side effects caused by taking antibiotics to other people (family, friends). |  |
| 14 | Combining antibiotics with traditional medicines can speed up disease recovery. |  |
| 15 | Taking antibiotics during pregnancy prevents miscarriage. |  |
| 16 | If a doctor prescribes medication, I can take them all without following the prescription. |  |
| 17 | Antibiotics are more effective than other medicines. |  |
| 18 | Medicines can be purchased anywhere. |  |
| 19 | After childbirth, antibiotics should be systematically given to women. |  |
| 20 | The duration of antibiotic use depends on the progress of disease recovery. |  |
| 21 | When the prescription is not readable, I take the medicine in my own way. |  |
| 22 | A pregnant or breastfeeding woman can take multiple antibiotics prescribed by a healthcare provider. |  |
| 23 | The treatment of pregnant women with antibiotics should be monitored by prescribers. |  |
| 24 | All medicine sellers should be trained on antibiotic prescriptions. |  |
| 25 | Drinking plenty of water helps reduce the possible negative effects of antibiotics. |  |
| 26 | Only qualified healthcare personnel should prescribe antibiotics to pregnant and breastfeeding women. |  |
| 27 | Antibiotics can always be requested during a consultation. |  |
| 28 | Always follow the advice of your health professional when using antibiotics. |  |
| 29 | I can share my antibiotics with other people. |  |
| 30 | I can use leftover antibiotics from a previous treatment if the symptoms seem the same. |  |
| 31 | Communities know how to use antibiotics properly. |  |
| 32 | Traditional medicines (leaves and herbs) can be used instead of antibiotics. |  |
| 33 | The use of antibiotics in pregnant women should only consider the health of the fetus. |  |
| 34 | All antibiotics can be prescribed to pregnant women regardless of the stage of pregnancy. |  |
| 35 | The dosage must be written in the mother’s health record. |  |
| 36 | I buy antibiotics directly without the advice of a health worker or without a prescription. |  |

**INTERVIEW GUIDE FOLLOWED TO Q SORTING:**

1. **Q-Method interview instructions**

Thank you for completing the ranking exercise. We will now discuss the statements you placed at the extremes — the three you strongly agreed with (+3) and the three you strongly disagreed with (–3). As a reminder, this interview is being recorded with your permission to help us better understand your views and reasoning. We will also ask a few follow-up questions to explore your personal reasoning, identify lived experiences, capture social and cultural influences, document community norms, and examine how perceptions of risk and healthcare are formed.

1. **Primary questions for each of the three statements that the participant strongly agreed with (+3) and the three they strongly disagreed with (–3)**

- Why did you place this statement at +3 / –3? Could you explain your choice?
- Was your opinion based on personal experience, something someone told you, or something observed in your community? Could you tell me more about it?
- How did this experience influence your opinion?
- Could you tell me more about that?
- What makes you feel this way about this statement?
- How do you think other people in your community see this issue?

1. **Supplementary clarification questions (To be used only when relevant to the participant’s response)**

- Have you ever taken more medicine than what was prescribed to you?
- Have you ever seen someone else misuse or take too much medication?
- How would you define an overdose in your own words?
- Do you currently own and use a maternal health booklet?
- When you visit a health facility, do health workers usually request to see it?
- Why do you think the maternal health booklet is important?
- What role does the maternal booklet play in your care?
- Have you ever not followed the recommended medication dose?
- Have you observed others taking medicine incorrectly or in excess?
- What comes to mind when you hear the word “overdose”?
- How would you explain what “dosage” means?
- Why is it important to follow the correct dosage?
- What might happen if someone does not respect the dosage?
- What information does a doctor usually write on a prescription?
- Why do you think some medicines require a prescription?
- Which medicines can be obtained without a prescription?
- In small local shops, is a prescription required to buy medicine?
- So some people can get medicine without a prescription — is that right?
