# Supplementary material for: Pregnant and breastfeeding women’s intention to follow medical advice before antibiotic use: a comparative pilot analysis using the theory of planned behavior in Mahajanga, Madagascar
Source: BMC Public Health. 2026 Jan 12;26:521. doi: 10.1186/s12889-026-26190-1 (PMC12888152; doi:10.1186/s12889-026-26190-1)
Supplement: Supplementary file 3 — Supplementary Material 3. [file 12889_2026_26190_MOESM3_ESM.docx]

**Supplementary results: Partial least squares regression (PLS-R) analysis**

**Quantitative assessment of internal consistency:** The internal consistency of the TPB constructs was estimated using Omega coefficients. Reliability values were relatively low for attitude (ω = 0.52), subjective norms (ω = 0.47), perceived behavioral control (ω = 0.39), and intention (ω = 0.33). Analytical robustness was reinforced through methodological triangulation combining multiple linear regression, logistic regression, and partial least squares regression (PLS-R), enabling cross-validation of findings across complementary statistical frameworks.

**Quantitative data analyses**

All the quantitative data analyses were conducted in R software (v.2025.05.0). Data from urban (Mahajanga I) and rural (Mahajanga II) participants were analyzed separately to capture context-specific variations.

**Sociodemographic analysis:** Associations between sociodemographic characteristics (age, marital status, education, employment, gravidity, breastfeeding status, timing and source of antibiotic use) and place of residence were tested with chi-square tests. A significance level of *p* ≤ 0.10 was used to flag potential trends, whereas *p* ≤ 0.05 was considered statistical significance.

**Multiple linear regression (MLR):** For each area, two models were estimated: Model 1, with TPB constructs, and Model 2, with sociodemographic covariates (age, gravidity, and household size) added. Multicollinearity was assessed using the Variance Inflation Factor (VIF); VIF > 5 indicated multicollinearity. Associations were evaluated using β coefficients, p values (≤ 0.05 significant), and explained variance (R²). Constructs with β ≥ 0.3 and significant p values were considered strong predictors.

**Binary logistic regression (BLR):** Behavioral intention (BI) scores, used as the outcome variable, were dichotomized (low/high, median split per site). Stepwise selection (backward AIC) identified the predictors. ATB, SN, and PBC were also dichotomized using the median split per site, and with the sociodemographic variables, they were used as explanatory variables for BI. All the explanatory variables included in the bivariate regressions were subsequently reintroduced into the multivariable regression. The results are reported as adjusted odds ratios (aORs) for multivariable analysis, with 95% CIs; *p* ≤ 0.05 indicated statistical significance. Predictors with aORs well above or below 1 were considered the most influential. Model fit was assessed with the Hosmer–Lemeshow test and discrimination with the (Area Under the ROC Curve).

**Partial least squares regression (PLS-R):** To address collinearity and small sample size, latent components of the TPB predictors are extracted. Model performance was assessed with the coefficient of determination (R²), representing the proportion of variance in the dependent variable (intention) explained by the model, and the Root Mean Square Error of Prediction (RMSEP), indicating the model’s predictive accuracy. The strength and direction of the relationships between predictors (Attitude, Subjective Norms, and Perceived behavioral control) and the dependent variable were assessed through standardized regression coefficients (β). A standardized β ≥ 0.3 with stable cross-validation indicated strong predictors.

**Partial least squares regression analysis of women’s intention to follow medical advice**

The partial least squares regression (PLS-R) highlights contextual contrasts in the determinants of intention. The first latent component model (Component 1) best summarizes the combined contribution of Attitude toward the behavior, Subjective norm, and Perceived behavioral control, providing the strongest prediction of women’s intention.

In urban areas, the PLS model with three components explained 29.22% of the variance in the intention. The component 1 explained about 26.67% of the variance (R² = 0.267), with a predictive correlation of 0.51. Subjective norms (β = 0.516) and perceived behavioral control (β = 0.343) outweighed attitudes (β = 0.062), underscoring the role of social influence and perceived ease of compliance.

In rural areas, the PLS model with three components explained 32.54% of the variance in the intention. The component 1 explained 27.27% of the variance (R² = 0.273), with a correlation of 0.52. Perceived control dominated (β = 0.644), followed by attitude (β = 0.192) and norms (β = 0.171), indicating that the ability to act matters more when resources are constrained.

The standardized coefficients reflected each variable’s weight, and the RMSEP values (between 0.5 and 0.7) confirmed good predictive quality. Overall, one-component models showed moderate explanatory power (R² values of 26.70% and 27.30%), although the drivers differed between urban and rural contexts [Table S1].

**Table S1.** Predictive performance and standardized coefficients of the PLS-R component

| **Predictor** | **Urban areas coefficient (β)** | **Rural areas coefficient (β)** |
| --- | --- | --- |
| **% Variance explained (R² des predictions) for women’s intention (Y) (on Component 1)** | 26.70% | 27.30% |
| **Root Mean Square Error of Prediction (RMSEP on component 1)** | 0.536 | 0.639 |
| **Attitude toward the behavior** | 0.062 | 0.192 |
| **Subjective norms** | 0.516 | 0.171 |
| **Perceived behavioral control** | 0.343 | 0.644 |

**Comparisons of multiple linear regression (MLR) and PLS-R results between urban and rural areas**
Using both multiple linear regression (MLR) and partial least squares regression (PLS-R) offered us robust complementary insight into the psychosocial determinants of women’s intention to follow medical advice. Despite their distinct frameworks, both approaches converged on similar patterns: in urban areas, subjective norms and perceived control were central, with attitude secondary; in rural areas, perceived control dominated, followed by attitude, while norms had a modest effect.

This convergence reinforces the robustness of the identified determinants, whereas the complementarity of both methods enriches the contextual interpretation. The MLR offers precise estimates of individual effects, whereas the PLS-R integrates psychosocial constructs through latent components and tolerates psychometric limitations. In both areas, the PLS-R explained approximately 27% of the variance in intention, with moderate correlations between the main latent component and intention (r = 0.52) [Table S2].

**Table S2.** Comparative summary of the MLR and PLS-R results by area

| **Model** | **Urban areas** | **Rural areas** |
| --- | --- | --- |
| **MLR – TPB Main predictor** | Subjective norms (β = 0.390) | Perceived Behavioral Control (β = 0.630) |
| **MLR – global R²** | 0.292 | 0.325 |
| **MLR – Other constructs contribution** | Perceived Control (β = 0.570) Attitude (β = 0.420) | Attitude (β = 0.66) Subjective Norm (β = 0.22) |
| **PLS-R – TPB main predictor** | Subjective norms (β = 0.516) | Perceived Behavioral Control (β = 0.644) |
| **PLS-R – Model R²** | 0.267 | 0.273 |
| **PLS-R – Other contributions** | Perceived Control (β = 0.343) Attitude (β = 0.062) | Attitude (β = 0.192) Subjective norms (β = 0.171) |
| **Correlation component–intention (PLS-R)** | r = 0.51 | r = 0.52 |
| **Complementarity** | Social influence central, practical capacity secondary | Practical capacity central, social influence secondary |
